# Class I histone deacetylase (HDAC) inhibitor CI-994 promotes functional recovery following spinal cord injury

**DOI:** 10.1038/s41419-018-0543-8

**Published:** 2018-04-27

**Authors:** Suxiang Zhang, Yuki Fujita, Rieko Matsuzaki, Toshihide Yamashita

**Affiliations:** 1 Department of Molecular Neuroscience, Graduate School of Medicine, Osaka University, 2-2, Yamadaoka, Suita, Osaka 565-0871 Japan; 2 WPI Immunology Frontier Research Center, Osaka University, Suita, Osaka 565-0871 Japan; 3 Graduate School of Frontier Biosciences, Osaka University, 1-3 Yamadaoka, Suita, Osaka 565-0871 Japan

## Abstract

Spinal cord injury (SCI) induces severe and long-lasting neurological disability. Accumulating evidence has suggested that histone deacetylase (HDAC) inhibitors exert neuroprotective effects against various insults and deficits in the central nervous system. In the present study, we assessed the effect of the class I HDAC inhibitor CI-994 in a mouse model of SCI. Following SCI, mice were treated with either dimethyl sulfoxide (control vehicle) or 1, 10, or 30 mg/kg CI-994. Level of acetylated histone H3 expression was increased in the motor cortex and spinal cord of 10 mg/kg CCI-994-treated mice after SCI. CI-994 increased histone H3 acetylation in the myeloperoxidase-positive neutrophils and CD68-positive microglia/macrophages in the spinal cord. Although it did not appear to contribute to corticospinal tract axonal reorganization, intraperitoneal injection of CI-994 promoted behavioral recovery following SCI. Furthermore, administration of CI-994 suppressed neutrophil accumulation, inflammatory cytokine expressions, and neuronal loss as early as 3 days following injury. Thus, our findings indicate that HDAC inhibitors may improve functional recovery following SCI, especially during the early stages of the disease.

## Introduction

Neuronal networks are destroyed by spinal cord injury (SCI), resulting in severe and long-lasting neurological disability. Microglia and inflammatory cells such as neutrophils and macrophages accumulate at the site of injury, leading to the release of the pro-inflammatory cytokines, proteases, and reactive oxygen species that cause tissue damage^1,2^.

Histone deacetylases (HDACs) belong to a family of enzymes that remove acetyl groups from lysine residues located on the amino-terminal tails of histone proteins, leading to the chromatin compaction associated with repression of transcription and reduced gene expression^3,4^. Based on their structure, HDACs can be classified as class I (HDAC1, 2, 3, and 8), class II (HDAC4, 5, 6, 7, and 9), class III (SIRT1–SIRT7), and class IV (HDAC11)^5^. Both class I and class II HDACs function via a zinc-dependent mechanism, whereas class III HDACs are NAD-dependent.

Accumulating evidence has suggested that HDAC expression was altered after injury of the central nervous system (CNS), and HDAC inhibitors exert neuroprotective effects against various insults and deficits in the CNS^6–8^. Previous studies have revealed that HDAC inhibitors reduce cortical neuronal cell death from ischemia and protect spinal motor neurons in vivo^9,10^. In addition, the HDAC inhibitor valproic acid (VPA) inhibits apoptosis following SCI by preventing blood–spinal cord barrier disruption and endoplasmic reticulum stress^11–13^. CI-994 (*N*-acetyldinaline) is a benzamide-based HDAC inhibitor that is relatively selective for class I HDACs^14^. CI-994 has at least a 100-fold greater preference for HDAC1 and HDAC3, relative to that for HDAC6 and HDAC8^15^. Previous evidence has also suggested that CI-994 attenuates fear memories^16^ and exerts neuroprotective effects both in vitro and in vivo^17,18^. However, the effects of CI-994 on SCI remain to be elucidated. In the present study, we demonstrated that CI-994 inhibits the accumulation of neutrophils in the injured spinal cord and reduces neuronal loss, and enhances functional recovery from SCI.

## Results

### HDAC1 and HDAC3 expression decreased 14 days after spinal cord injury

**Fig. 1:**
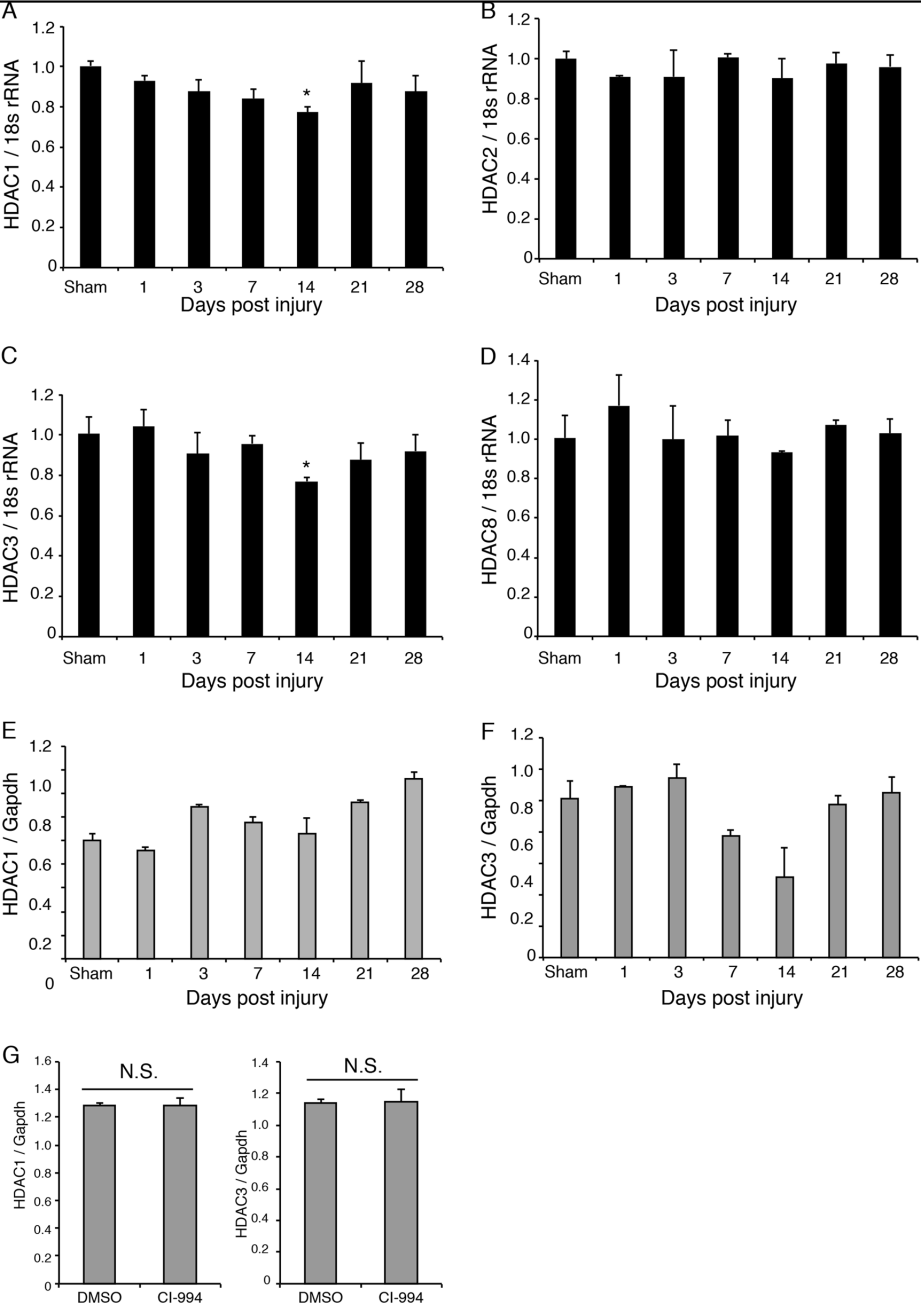
Changes in HDAC expression following SCI. **a**–**f** Profiles of HDAC expression in the motor cortex **a**–**d** and spinal cord **e**, **f** following SCI. Levels of class I HDAC expression relative to those of GAPDH were measured via real-time PCR. Results represent the mean ± SE from three independent experiments. Relative levels of HDAC expression are presented as fold changes relative to the level in sham mice. *n* = 3. *p < 0.05; ANOVA with Tukey–Kramer test. **g** HDAC expression was not altered in the spinal cord of CI-994-treated mice compared with DMSO-treated control mice. HDAC1 and three expressions were examined 3 days after SCI. *n* = 3. N.S., not significant; Student’s t-test. HDAC: histone deacetylase; SCI: spinal cord injury; GAPDH: Glyceraldehyde 3-phosphate dehydrogenase

**Fig. 2:**
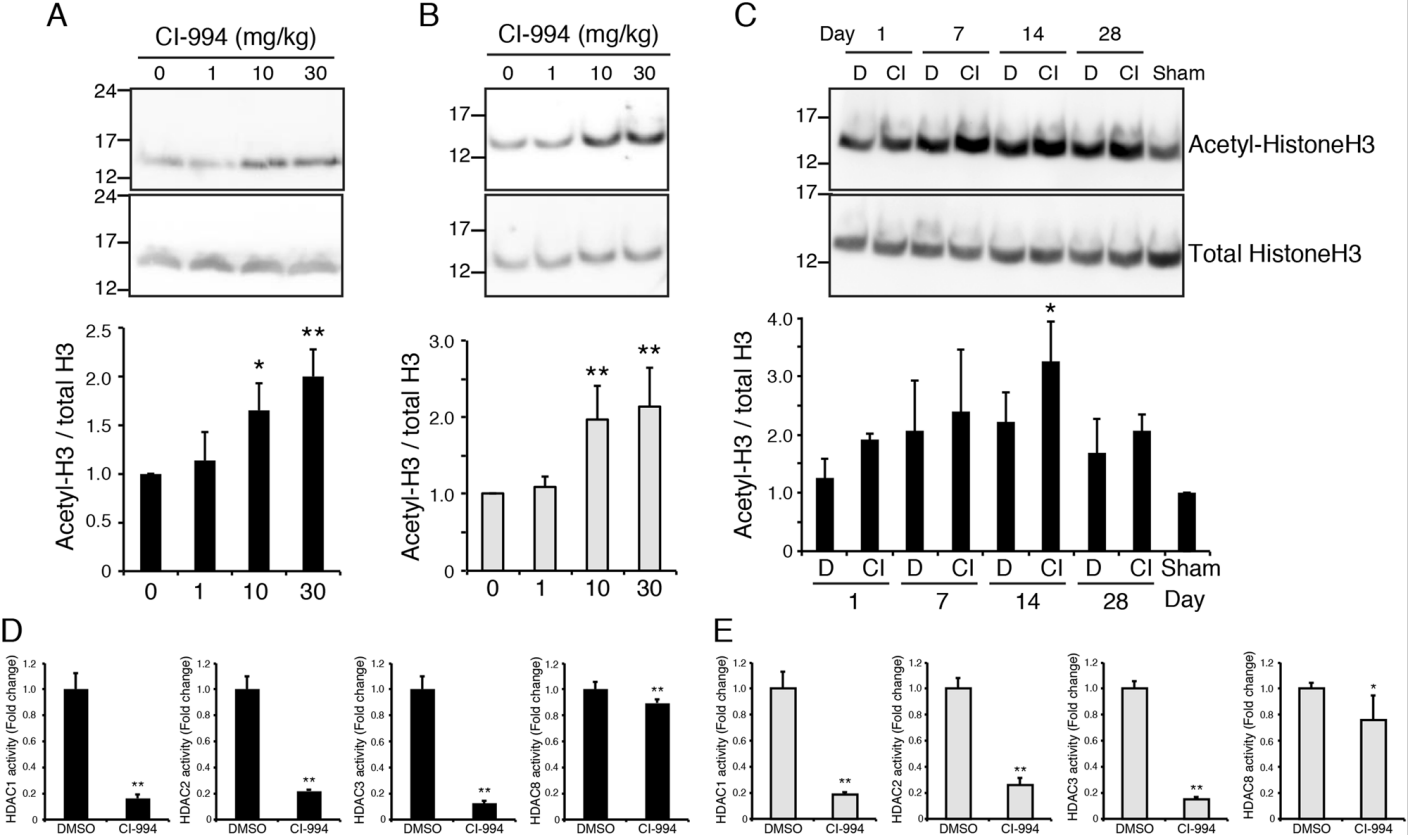
CI-994 increases HDAC activity after SCI. **a**, **b** Histone H3 acetylation was upregulated in the motor cortex **a** and spinal cord **b** of CI-994-treated mice. Western blot analysis revealed that 10 or 30 mg/kg CI-994 treatment once a day for 14 days tended to increase acetylated histone-H3 levels. *n* = 4. **p < 0.01, *p < 0.05; ANOVA with Tukey–Kramer test. **c** Time-course of histone H3 acetylation in the motor cortex. Mice were administrated with DMSO or 10 mg/kg CI-994 after SCI. *n* = 3. *p < 0.05; ANOVA with Tukey–Kramer test. **d**, **e** Class 1 HDAC activities were reduced in the motor cortex **d** and spinal cord **e** of CI-994-treated mice. *n* = 6. **p < 0.01, *p < 0.05; Student’s *t*-test

We first examined whether the expression of Class I HDACs was affected by the spinal cord injury (SCI). As our SCI model mice exhibited impaired hindlimb motor function, we focused on such changes in the motor cortex. Among class I HDACs, the levels of HDAC1 and HDAC3 expression were significantly decreased in the motor cortex 14 days after SCI (Fig. 1a–d), suggesting that both are involved in the physiological processes that occur following SCI. HDAC expression was not significantly changed in the lesion site of the spinal cord (Fig. 1e, f). To inhibit HDAC activity, we employed the class I HDAC inhibitor CI-994, which demonstrates a preference for HDAC1 and HDAC3 relative to HDAC8 (*K*_i_ values: 0.41 μM for HDAC1, 0.75 μM for HDAC3, and 100 μM for HDAC8, respectively)^15^. No expression changes in HDAC1 and HDAC3 were detected in the spinal cord of CI-994-treated mice (Fig. 1g) We therefore examined the acetylation level of histone H3 in the motor cortex and spinal cord, and evaluated the efficacy of CI-994 in SCI model mice. Following SCI, mice were treated with either dimethyl sulfoxide (DMSO) (control vehicle) or 1, 10, or 30 mg/kg CI-994, and the motor cortex or spinal cord tissues were subjected to Western blot analysis using anti-acetyl-histone H3 antibody. Mice treated with either 10 or 30 mg/kg CI-994 exhibited increased acetylation of histone H3 relative to that observed in vehicle-treated mice (Fig. 2a, b). These results indicated that 10 mg/kg CI-994 is sufficient for the inhibition of HDAC activity in the motor cortex (Fig. 2a) and spinal cord (Fig. 2b) of SCI model mice. We also examined the time-course of changes in histone acetylation. Increased acetylation of histone H3 was observed in the cortex of mice treated with 10 mg/kg CI-994, especially 14 days after SCI (Fig. 2c).

We further examined whether CI-994 inhibits HDAC activity in vivo by using an immunoprecipitation-based HDAC activity assay kit. Tissues from cortex and spinal cord were prepared from mice at 14 day or 3 day after SCI, respectively. Administration of CI-994 inhibited class I HDAC activities in both the cortex and spinal cord (Fig. 2d, e). The inhibitory effect of CI-994 on HDAC8 activity was weaker than that on other class 1 HDACs. These results indicate that CI-994 increases the acetylation level of histone H3 by inhibiting class I HDACs in the SCI model, consistent with the findings of previous in vivo and in vitro studies^16,19^.

**Fig. 3:**
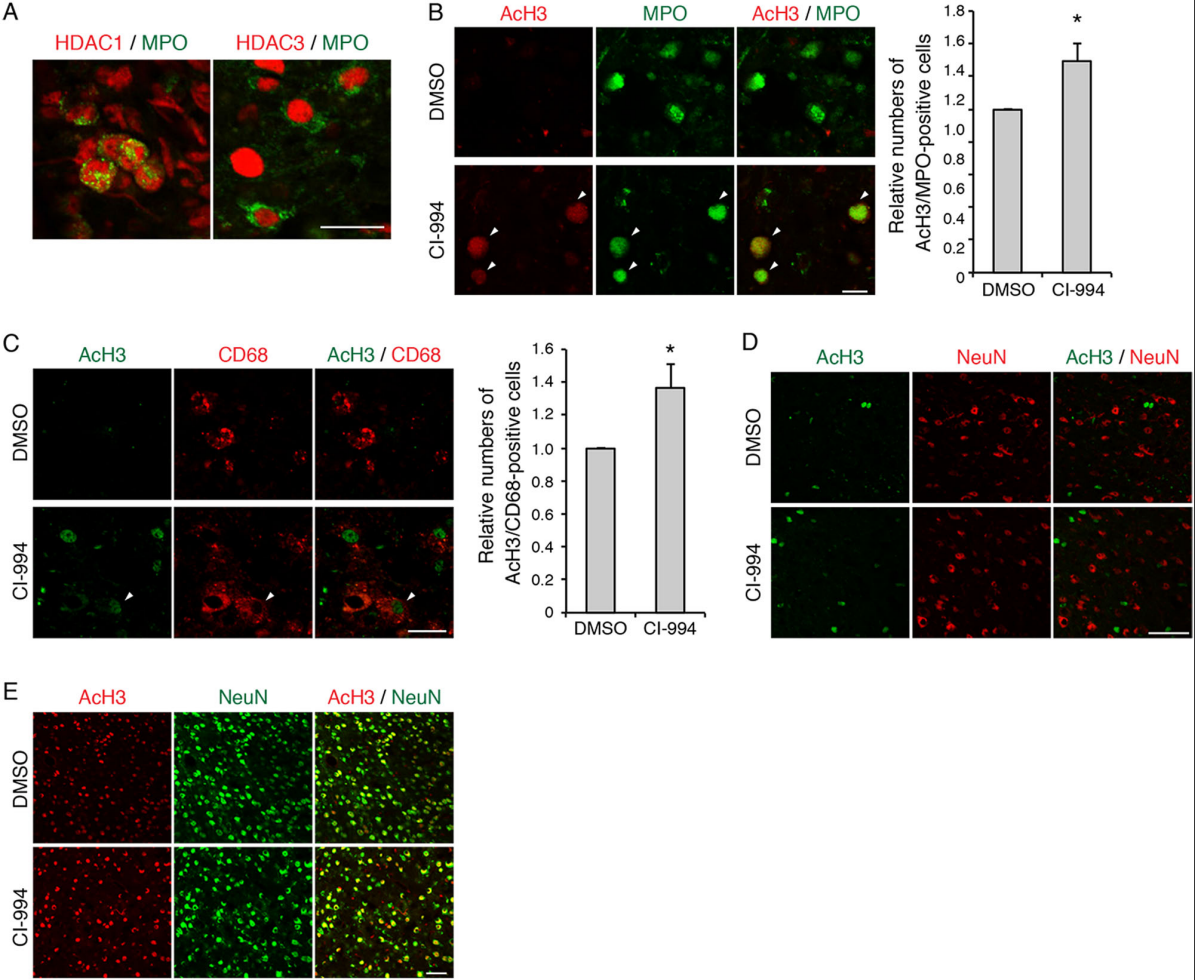
CI-994 increases acetylated histone H3 level in immune cells. **a** HDAC1 and HDAC3 expression was observed in MPO-positive cells from 3 day after SCI. Scale bar 20 μm. **b**–**e** Double-staining images of acetylated histone H3 and indicated cell-type specific markers in the spinal cord **b**–**d** and cortex **e**. Increased acetylated histone H3 in MPO-positive cells (Green in **b**) and CD68-positive cells (Red in **c**) in the spinal cord of CI-994-treated mice. Arrowheads: co-labeling of anti-acetylated histone H3 and cell-type specific markers. Scale bars 20 μm in the spinal cord images **b**–**d**; 50 μm in the cortex images **e**. *n* = 6. *p < 0.05; Student’s *t*-test. HDAC: histone deacetylase; SCI: spinal cord injury

As immune cells such as neutrophils and microglia/macrophages have been shown to accumulate around lesion site after SCI, we examined the HDAC activity in these cells. Expression of HDAC1 as well as HDAC3 was observed in MPO-positive neutrophils in the spinal cord from 3 day after SCI (Fig. 3a). Myeloperoxidase (MPO)-positive neutrophils and CD68-positive microglia/macrophages in the spinal cord showed increased co-labeling of acetylated histone H3 in the CI-994-treated mice compared to controls (Fig. 3b, c). When NeuN-positive neurons were co-immunostained with acetylated histone H3, co-labeling was detected in the cortex but not in the spinal cord (Fig. 3d, e). These results suggest that administration of CI-994 reduces HDAC activity and increases acetylation of histone H3 in the cortex and spinal cord.

### CI-994 enhances functional recovery following SCI

**Fig. 4:**
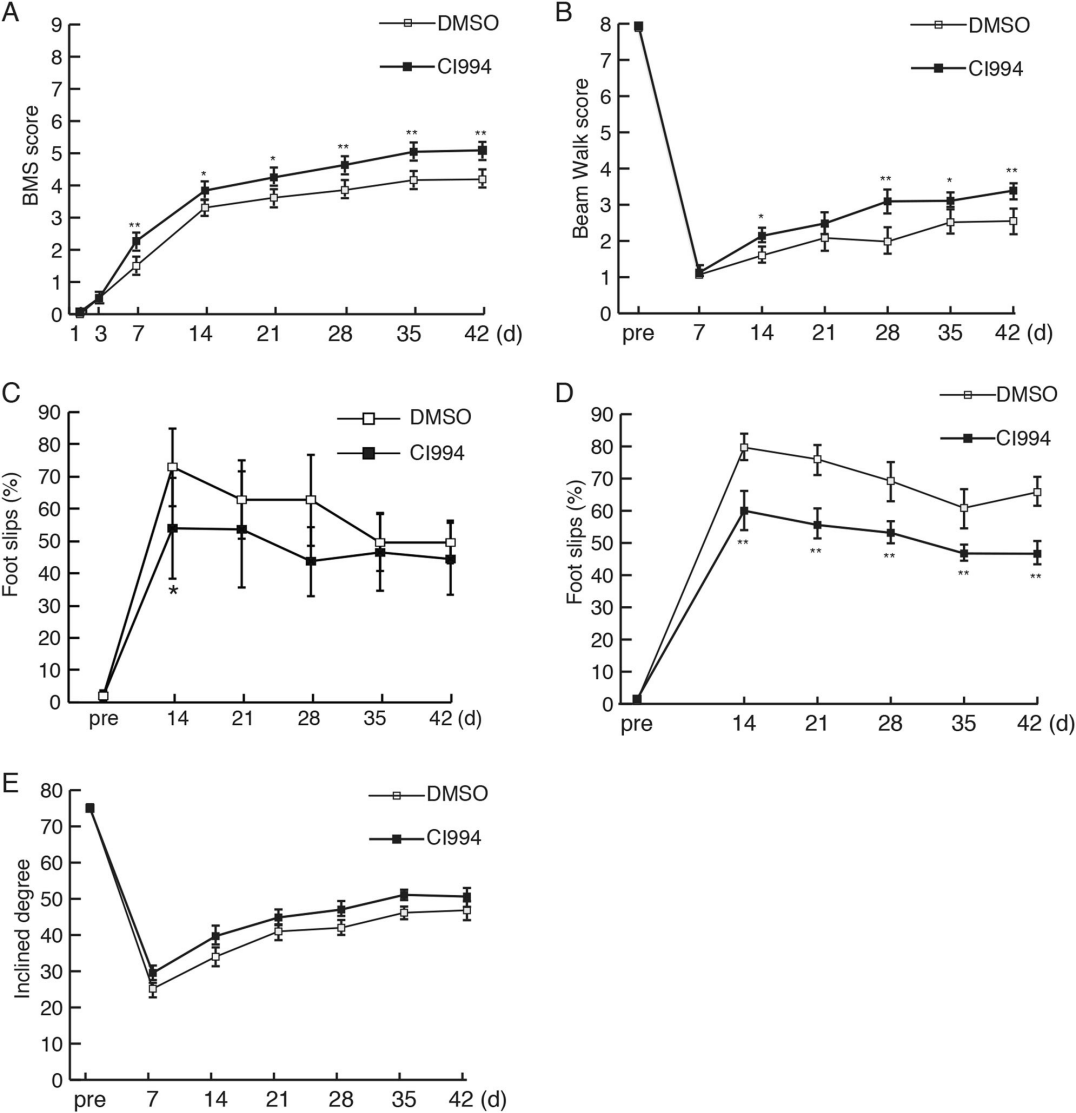
CI-994 treatment improves recovery of motor function following SCI. **a** BMS scores after injury were significantly increased in CI-994-treated mice relative to those in vehicle (DMSO)-treated mice. **b** CI-994 treatment attenuated increases in the number of foot-slips in the beam walk test after SCI. **c** CI-994 treatment attenuated increases in the number of foot-falls in the grid walk test after SCI. **d** CI-994 treatment reduced the percentage and number of errors in the ladder walk test. **e** There was no significant difference among the groups in the inclined plane test. Results are presented as the mean ± SE. DMSO *n* = 21, CI-994 *n* = 22 in **a**; DMSO *n* = 15, CI-994 *n* = 16 in **b**–**e**. *p *<* 0.05, **p *<* 0.01. Two-way repeated-measures ANOVA with Tukey–Kramer test. SCI: spinal cord injury; BMS: Basso Mouse Scale

We next investigated whether intraperitoneal injection of CI-994 promoted behavioral recovery following SCI. As thoracic spinal cord injury mainly impaired motor function in the descending level of the lesion site, we evaluated the motor function, especially in the hindlimbs. We first used the Basso Mouse Scale for locomotion (BMS), which is widely used to evaluate locomotor recovery following SCI^20^. Both vehicle- and CI-994-treated mice exhibited impaired BMS score 1 day after SCI (Fig. 4a). The score gradually increased over 4 weeks, suggesting some degree of spontaneous recovery from the neurological deficit. Seven days after SCI, CI-994-treated mice exhibited higher BMS scores than controls (Fig. 4a). To verify this result, we further assessed the functional recovery of the hindlimbs using other motor tests. The footfall frequency of the hindlimbs during walking was assessed using the beam walking, grid walking, and ladder-walking tests. CI-994-treated mice exhibited improved functional recovery relative to control mice at 14 days after SCI (Fig. 4b–d). Weight support and coordination between forelimbs and hindlimbs were assessed via the inclined plane test. However, no significant differences were detected between the two groups (Fig. 4e). These results indicate that CI-994 promotes functional recovery in the early stages following SCI.

### CI-994 treatment does not enhance cervical sprouting of the corticospinal tract (CST) following SCI

**Fig. 5:**
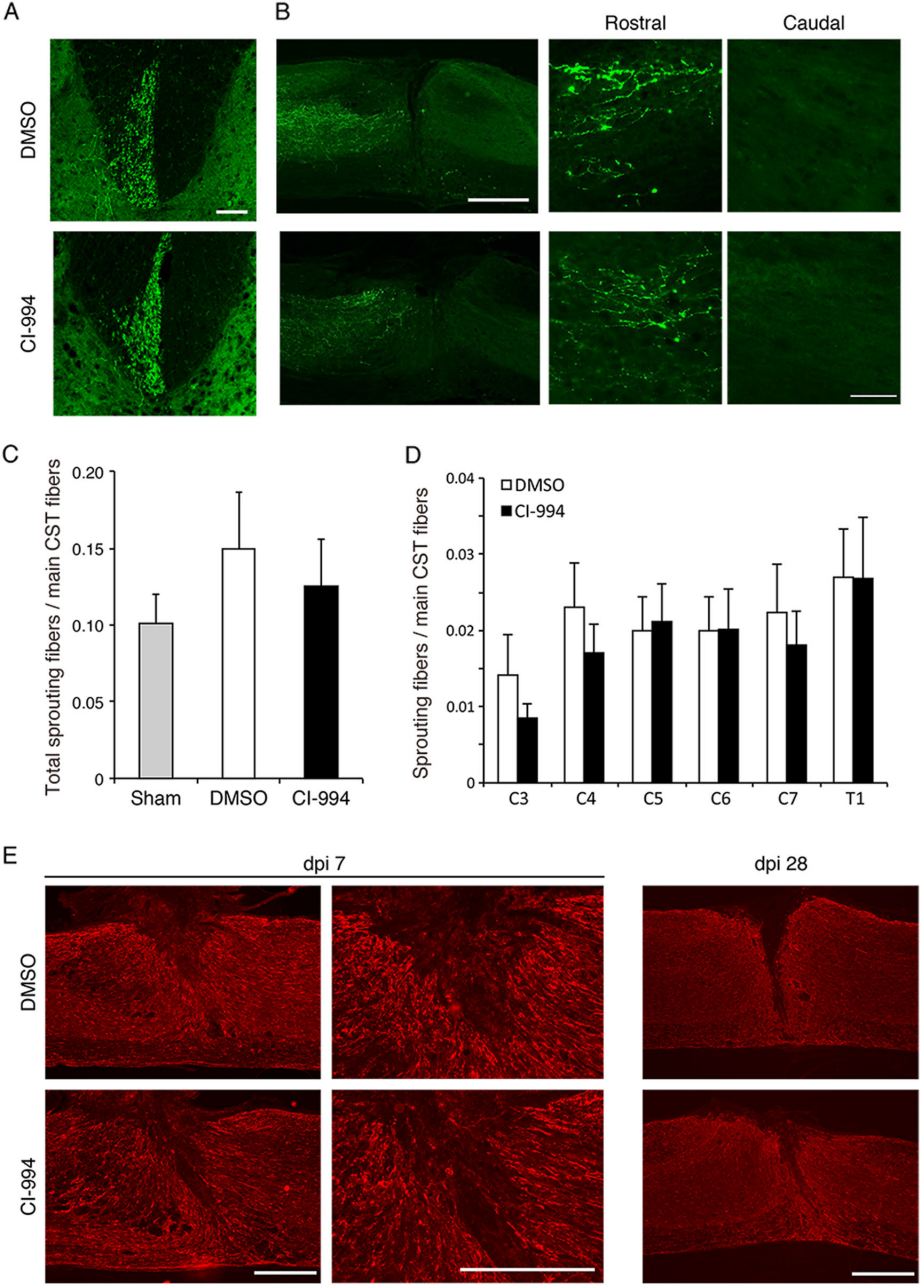
CI-994 does not promote sprouting of corticospinal tract fibers following SCI. **a**, **b** Representative images from the transverse sections of the cervical spinal cord **a** and sagittal sections of the lesion site, which show biotinylated dextran amine (BDA)-labeled corticospinal tract fibers (green) extending into the gray matter at 28 days following injury. Scale bar 100 μm (**a**, high magnification images in **b**), 200 μm (low-magnification images in **b**). **c**, **d** The ratio of the total number of sprouting corticospinal tract fibers **c**, or the number of the fibers at the indicated cervical or thoracic spinal cord levels **d** at 28 days after injury. No significant differences were observed between control (DMSO) and CI-994-treated mice. Student’s *t*-test. Sham: *n* = 7; DMSO, CI-994: *n* = 14. **e** Regions of reactive gliosis were visualized as GFAP-positive areas near the site of injury in control or CI-994-treated mice. dpi: days post injury. Scale bar 200 μm. SCI: spinal cord injury; GFAP: glial fibrillary acidic protein

Previous studies have reported that SCI mice subjected to hemi-section of the thoracic cord exhibit fiber sprouting from axons of the CST at the level of the cervical cord, which contribute to rewiring of the neuronal network and functional recovery^21^. These findings indicate that increased sprouting of CST axons promotes functional recovery, and that class I HDACs may be involved in restricting axonal plasticity following SCI. Thus, we assessed whether inhibition of HDAC promotes axonal sprouting after SCI. Following SCI, mice were treated with either 10 mg/kg CI-994 or control vehicle once per day. Two weeks after surgery, mice were injected with the anterograde tracer, biotinylated dextran amine (BDA), for labeling of CST axons. The number of sprouting fibers extending from the CST into the gray matter was normalized by the intensity of the main CST and analyzed 4 weeks after SCI. There was no significant difference in the relative number of sprouting fibers between control and CI-994-treated mice (Fig. 5a–d). In the lesion site, sagittal sections showed no differences in the BDA-labeled fibers in the rostral side, and the fibers were not observed in the caudal side, suggesting that CI-994 did not induce axonal regeneration (Fig. 5b). We also examined differences in gliosis between the two groups. glial fibrillary acidic protein (GFAP)-staining of reactive astrocytes confirmed that there was no difference in the volume of spinal cord lesions between control and CI-994-treated mice (Fig. 5e). These results suggest that CI-994 does not affect axonal branching following SCI.

### Neutrophil infiltration is decreased in CI-994-treated mice

**Fig. 6:**
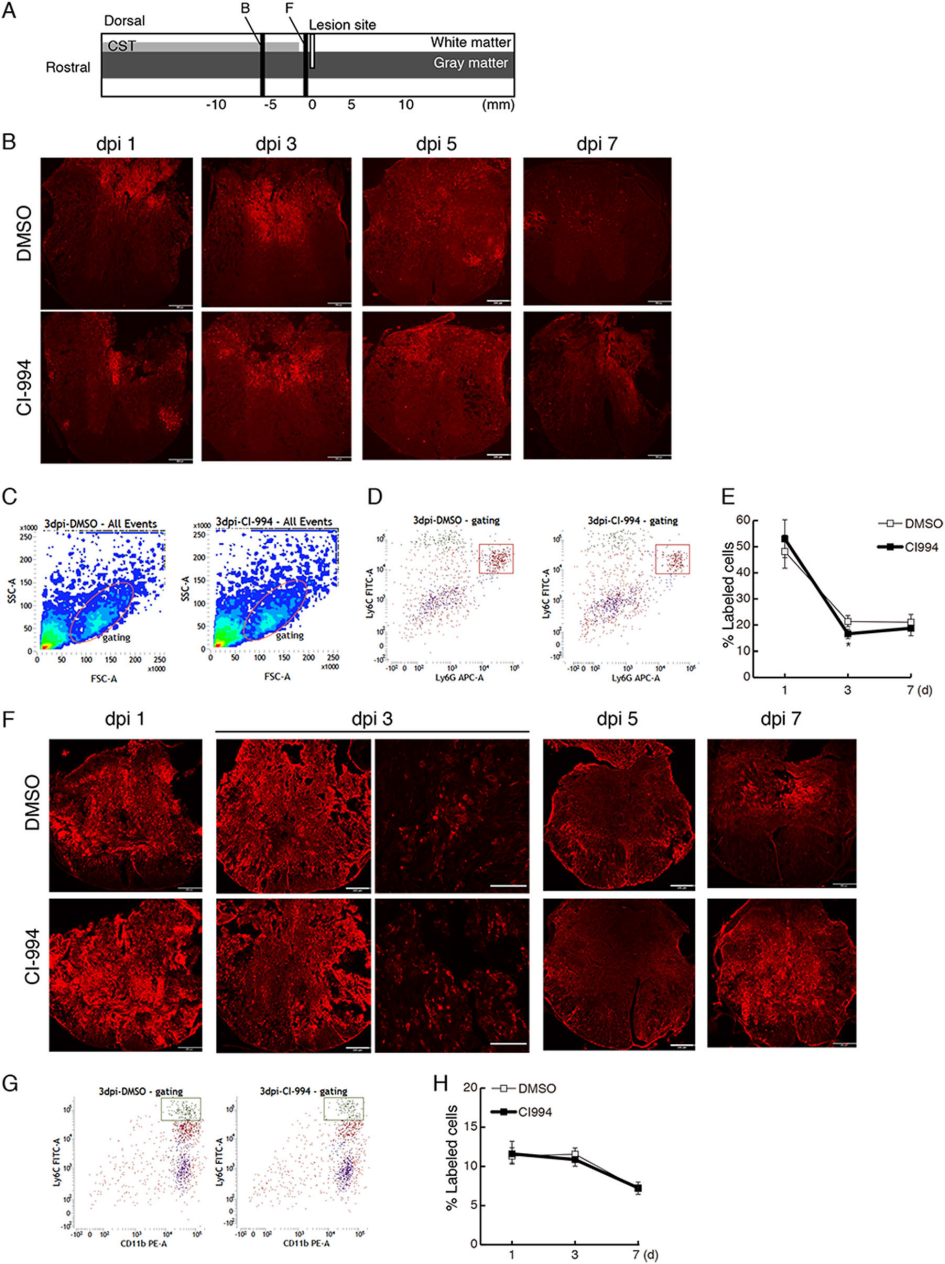
CI-994 reduces neutrophil infiltration following SCI. **a** Schematic representation of injured spinal cord. Transverse sections were prepared from 6 mm rostral **b**, or adjacent to the lesion site (F). **b** The number of MPO-positive neutrophils was decreased in the injured spinal cord of CI-994-treated mice at 1 day and 3 days after SCI. dpi: days post injury. Scale bar: 200 μm. **c** Dot plots of isolated immune cells in the spinal cord, gated for live cell analysis. **d** Representative cytometry data for neutrophils (Ly6C^+^/Ly6G^+^ cells) in the injured spinal cord 3 days after SCI. **e** Quantitative data for accumulated neutrophils in the injured spinal cord up to 7 days after SCI. **f** Numbers of CD68 (ED-1)-positive activated microglia/macrophages were not altered in the injured spinal cord of CI-994-treated mice. Scale bar: 200 μm in low-magnification images, 100 μm in high magnification images. **g** Representative cytometry data for macrophages (Ly6C^+^/CD11b^+^ cells) in the injured spinal cord at the indicated days after SCI. **h**Quantitative data for accumulated macrophages in the injured spinal cord up to 7 days after SCI. Results are presented as the mean ± SE (*n* = 3–6). *p *<* 0.05, Two-way repeated-measures ANOVA. SCI: spinal cord injury; MPO: myeloperoxidase

As CI-994 seemed to enhance functional recovery without promoting axonal reorganization, we hypothesized that CI-994 suppresses inflammation in the acute to sub-acute phases following SCI. As a robust inflammatory response occurs following SCI, we first examined the time-course of immune cell accumulation in the spinal cord until 1 week after SCI. Transverse sections of the spinal cord obtained from 6 mm rostral or adjacent to the rostral lesion site were subjected to immunostaining (Fig. 6a). Although numerous MPO-positive neutrophils were observed around the site of injury 1 day after SCI (Fig. 6b), their number gradually decreased over time such that most MPO-positive cells had disappeared by 7 days after injury. These observations are consistent with those reported in previous studies^22^. Levels of Ly6C- and Ly6G-positive neutrophils were increased in ~ 48% of gated immune cells at 1 day after injury, though levels decreased by 7 days post injury (Fig. 6c–e). CI-994 treatment suppressed accumulation of neutrophils relative to the levels observed in vehicle-treated mice 3 days post injury (Fig. 6e).

Numerous CD68 (ED-1)-positive activated microglia/macrophages were observed around the lesion site 1 day after injury (Fig. 6f). However, there were no significant differences in the percentage of macrophages (Ly6C^high^/CD11b^+^ cells) between CI-994-treated and control mice (Fig. 6g, h).

### CI-994 exerts neuroprotective effects after SCI

**Fig. 7:**
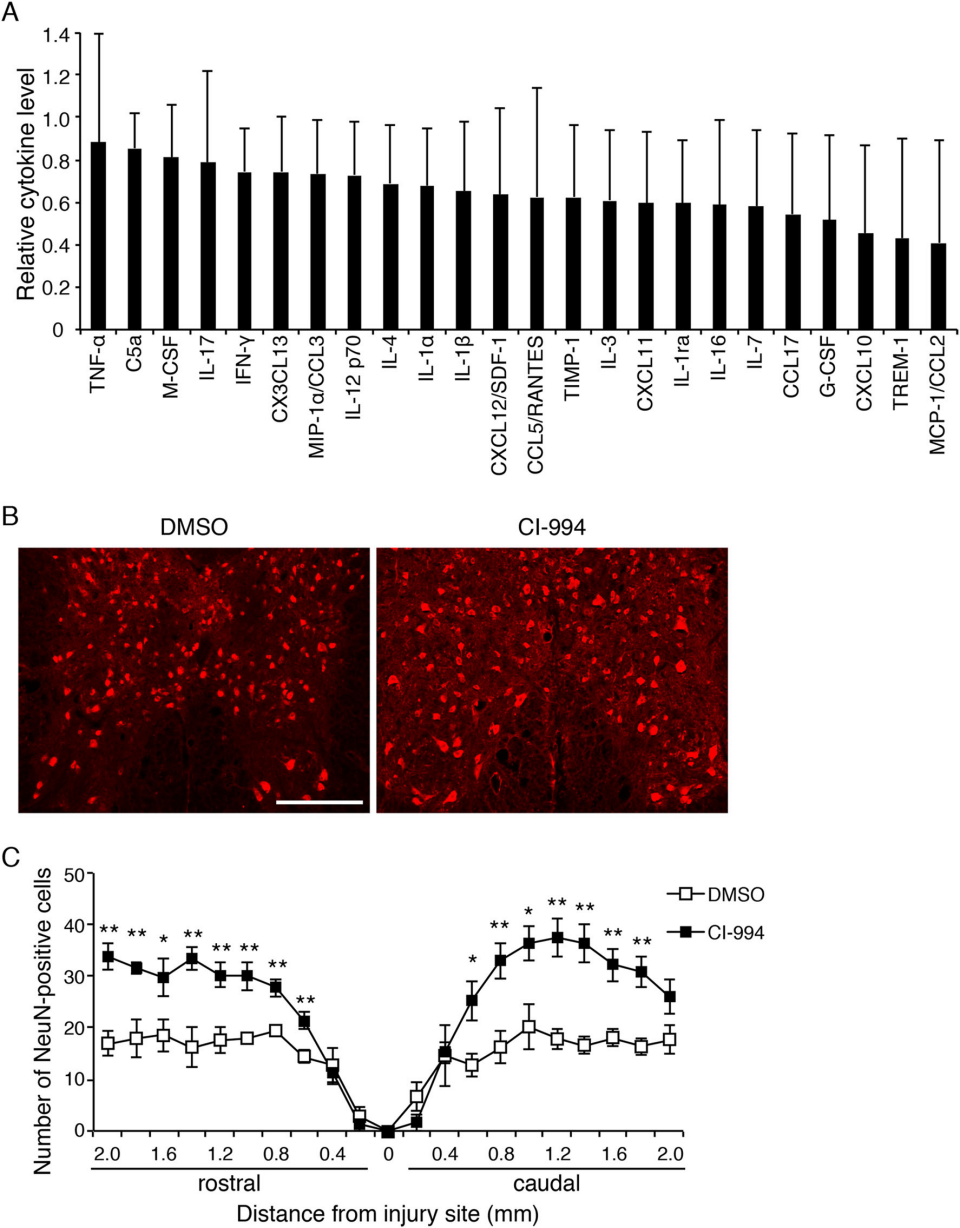
CI-994 ameliorates the loss of NeuN-positive neurons following SCI. **a** Decreased cytokine/chemokine expression in the spinal cord of CI-994-treated mice. Cytokine/chemokine levels in CI-994-treated mice were normalized to the levels in DMSO (control)-treated mice. *n* = 4. **b** Representative images of immunohistochemical staining of neurons in the gray mater following SCI. Neurons were immunostained with anti-NeuN antibody. Scale bar: 200 μm. **c** The numbers of neurons in the gray matter from 2 mm rostral to lesioned sites to 2 mm caudal to lesioned sites were calculated at the indicated days after SCI. Results are presented as the mean ± SE of five mice per group. *p *<* 0.05, **p *<* 0.01; ANOVA with Tukey–Kramer test. SCI: spinal cord injury

The infiltration of immune cells leads to neurotoxic damage associated with the release of inflammatory molecules such as reactive oxygen species and free radicals^1,23^. To investigate whether CI-994 suppresses the inflammatory response after SCI, we examined the cytokine levels in the lesion site of the spinal cord using a cytokine/chemokine array. We found that inflammatory cytokines, such as tumor necrosis factor-α (TNF-α) and interferon-γ (IFN-γ), seemed to be decreased in CI-994-treated mice compared with vehicle-treated mice (Fig. 7a). Monocyte chemoattractant protein-1 (MCP-1/CCL2), which regulates the infiltration of monocytes/macrophages, also tended to be decreased. The expression of the triggering receptor expressed on myeloid cells, which is the immunoglobulin receptor in neutrophils and monocytes related to the amplification of the acute inflammatory response, was also repressed.

As our findings support that CI-994 reduces inflammatory responses, we further investigated whether CI-994 also suppresses the loss of neurons after SCI. As motor function recovery was observed from 7 days after SCI (Fig. 4a) and neutrophil infiltration was suppressed 3 days after SCI (Fig. 6e), we focused on the effect of CI-994 on neurons in the early phase after SCI. CI-994-treated mice exhibited a significantly greater number of NeuN-positive cells than control mice 3 days after SCI (Fig. 7b,c). These results suggest that CI-994 exerts neuroprotective effects after SCI.

## Discussion

In the present study, we demonstrated that the class I HDAC inhibitor CI-994 significantly improved recovery of motor function following SCI. Furthermore, administration of CI-994 suppressed neutrophil accumulation and neuronal loss at the site of injury. Thus, our findings indicate that inhibition of HDAC activity is important for neuronal protection and recovery following SCI.

We observed that levels of HDAC1 and HDAC3 expression were reduced in the motor cortex at 14 days after SCI, at which time increases in CST sprouting were also observed^24,25^. These findings indicate that HDAC1 and HDAC3 may be correlated with the spontaneous sprouting of CST fibers following SCI. However, the inhibition of HDAC1 and HDAC3 by CI-994 did not promote axonal rewiring after SCI (Fig. 5), suggesting that CI-994 promotes functional recovery through other mechanisms. Previous findings have indicated that HDAC inhibitors exert neuroprotective effects associated with blockade of the inflammatory response^10,26,27^. Furthermore, administration of the HDAC inhibitor, VPA, inhibits neuronal cell death after SCI^9^. Our findings regarding the neuroprotective effect of CI-994 are consistent with these previous results, supporting the notion that CI-994 suppresses inflammation and prevents the loss of neurons after SCI.

Behavioral outcomes following SCI can be evaluated using various methods^28,29^. In the present study, we used five behavioral tests to assess the effect of CI-994 after injury. CI-994 promoted functional recovery as indicated by the BMS, beam walk, grid walk, and ladder walk tests, although no improvements were noted with regard to the inclined plane test (Fig. 4). The beam walk, grid walk, and ladder walk tests are considered to reflect the integrity of descending motor control^28^, indicating that results on these tests accurately depict the recovery of motor function induced by CI-994 treatment. However, the inclined plane test evaluates the function of skeletal muscles not predominantly involved in locomotion and thus does not detect the effects of CI-994. Rather, the results of this test correlate with the integrity of non-pyramidal pathways (e.g., the rubrospinal tract) following SCI^30^. Taken together, these findings indicate that CI-994 may contribute to functional recovery by supporting regeneration in the pyramidal tract.

In the present study, CI-994 significantly inhibited neutrophil accumulation and neuronal loss, both of which are increased after SCI (Figs. 6 and 7). Therefore, our results suggest that CI-994 exerts neuroprotective effects in part via inhibition of neutrophil accumulation, leading to reductions in the level of inflammatory cytokines after SCI. Inhibition of HDAC activity has been shown to reduce the inflammatory processes in many animal models of neurological disease, such as traumatic brain injury and age-associated memory impairment^31,32^. Our results demonstrate that the expression levels of classical inflammatory cytokines, such as TNF-α, are decreased by CI-994 treatment (Fig. 7a). Although we did not detect significant differences in the infiltration of microglia/macrophages by immunohistochemistry or fluorescence-activated cell sorting (FACS) analysis, the expression of chemokines, such as macrophage inflammatory proteins-1 α, which is produced by these cells and has an important role in inflammation, decreased in CI-994-treated mice. These results also suggest that CI-994 reduces inflammatory responses not only by the suppression of neutrophil accumulation but also by the negative modulation of macrophages. Since the acetylated histone H3 was increased in MPO-positive and CD68-positive cells but not NeuN-positive neurons, cell-type-specific expression of cytokines will provide greater differences.

A recent study reported that the specific HDAC3 inhibitor suppresses inflammation after SCI, whereas axonal growth was not directly affected by the inhibition of HDAC3^33^. Consistent with the findings of the study, our results showed that CI-994 reduced inflammatory cytokine expressions, suggesting that the effect of CI-994 may predominantly depend on its ability for HDAC3 inhibition. Although the accumulation of CD68-positive macrophages was not significantly changed in our study, there still remains the possibility that CI-994 affects the innate immunity. As inhibition of HDAC3 is considered to shift microglia/macrophage state toward inflammatory suppression, it is intriguing to hypothesize that CI-994 reduced the reactive state of microglia/macrophages. We further demonstrated that administration of CI-994 increased the number of neurons around the lesion site (Fig. 7b, c). CI-994 also inhibited HDAC1 (Fig. 2d, e). It has been reported that inhibition of HDAC1 contributes to neuroprotective function^34^. Thus, inhibition of both HDAC1 and 3 of CI-994 may exert neuroprotective effect.

The other benzamide-based HDAC inhibitor, MS-275, induces the expression of genes associated with axon regeneration in sensory neurons and promotes axonal growth after the axotomy of peripheral neurons^35^. Compared with neurons in the CNS, peripheral neurons have higher potentials for intrinsic axon growth. Although we could not assess the effect of CI-994 on the peripheral nervous system (PNS), it is possible that the regenerative capability of HDAC inhibitors differs between the CNS and the PNS.

In summary, our findings showed that the class I HDAC inhibitor, CI-994, exerted neuroprotective effects in a mouse model of SCI. At present, several HDAC inhibitors are under investigation in clinical trials for cancer treatment, such as the benzamide-based HDAC inhibitors Chidamide, CI-994, and MS-275^36,37^. Therefore, future studies should aim to elucidate the precise mechanisms underlying the effects of CI-994 on SCI.

## Materials and Methods

### Mice

Eight-week-old C57BL/6 J mice obtained from Japan SLC, Inc. (Shizuoka, Japan) were bred and maintained at the Institute of Experimental Animal Sciences, Osaka University Graduate School of Medicine. This study was approved by the institutional committee of Osaka University, and all experiments were performed in accordance with the Guide for the Care and Use of Laboratory Animals of the Osaka University Medical School.

### Surgical procedures for SCI and CI-994 treatment

Mice were anesthetized with a mixture of butorphanol (Vetorphale®, 0.5 mg/ml, Meiji Seika Pharma, Tokyo, Japan), midazolam, (Dormicum®, 0.4 mg/ml, Roche), and medetomidine (Domitor®, 0.03 mg/ml, Orion Pharma) via peritoneal injection. SCI was performed as previously described^38^. In brief, the connective and muscle tissues were removed to expose the lower thoracic spinal cord. Following T8 laminectomy, dorsal hemi-section was performed at T8 using a surgical blade at a depth of 1.0 mm. Avoiding the remaining portion of the lateral CST, the surgical blade was passed through the dorsal spinal cord several times to create a lesion that extended downward to the central canal. All mice showed complete paralysis of both hindlimbs after surgery.

CI-994 (4-Acetamido-N-(2-aminophenyl) benzamide Tokyo Chemical Industry, JAPAN) was administered at 3 h after injury and then once daily for 14 days via intraperitoneal injection. CI-994 was dissolved in the vehicle (DMSO) and administered at the indicated concentrations (0, 1, 10, or 30 mg/kg). CI-994 (10 mg/kg) was administered following SCI, unless otherwise indicated.

### Behavioral tests

All animals were subjected to five motor tests for the evaluation of functional recovery after SCI: the BMS, beam walk, grid walk, ladder walk, and inclined plane tests. To evaluate normal performance, each animal’s baseline score was recorded just before the surgery. For the beam walking and ladder walk tests, mice were trained to walk on a narrow beam or on a horizontal ladder prior to injury, respectively.

### BMS score

Previous studies have indicated that the BMS score is correlated with hindlimb motor function^20^. BMS scores were determined at the following time points: 1, 3, 7, 14, 21, 28, 35, and 42 days after injury. The average scores of the right and left hindlimbs were used.

### Beam walking test

A modified version of the beam walking test was employed to assess the severity of motor incoordination^28,39^. Narrow wood beams (width: 1.4 cm; length: 100 cm; height from ground: 15 cm) were employed, and scores were determined as previously described^38^. At 7, 14, 21, 28, 35, and 42 days after injury, each mouse went through the beam four times and the average scores were recorded.

### Grid walk test

The grid walk test was used to evaluate the ability to accurately place the hindpaws on the rungs of a grid during spontaneous exploration^40,41^. Mice were placed on a wire grid (200 × 240 mm) with 12-mm square holes and allowed to freely explore for 5 min, while performance was recorded with a video camera. Foot-slip denotes that the paw completely missed a rung or despite the paw was correctly placed on a rung but slipped off when supporting the body weight. The percentage of foot-slips within 50 steps for each hindpaw was calculated, and the average percentages of the right and left hindpaws were recorded at 14, 21, 28, 35, and 42 days after injury.

### Ladder walk test

The ladder walk test was used to assess precise limb placement and stepping while walking along a horizontal ladder with variable rung space^42^. The ladder was designed as previously described^43^. Mice received training three times per session the day before the injury and the percentage of foot-slips of each hindpaw was recorded. The tests started at 2 weeks after injury and then performed once a week for additional 4 weeks. The average scores of the right and left hindlimbs were used.

### Inclined plane test

The inclined plane test was used to assess the ability of a mouse to maintain its body position on a sloping board covered with rubber. The method was modified based on the protocol used in a previous study^44^. The test was performed in two positions (right-side or left-side up), and the angle of board was gradually increased toward the vertical position. The greatest angle at which a mouse could maintain its stable position for 5 s without falling was recorded, and the angles in two positions were averaged to obtain a single score for each mouse.

### Anterograde CST labeling

Two weeks after surgery, mice were anesthetized as previously described and placed on a stereotaxic frame. The skull was carefully split open using a drill. To visualize the uninjured CST, the anterograde tracer BDA (molecular weight: 10000; 10% in phosphate-buffered saline (PBS); Invitrogen, Carlsbad, CA, USA) was injected at three sites within the hindlimb motor area (three sites; 0.6 µl per site) using a 5-μl microsyringe (Ito, Shizuoka, Japan) with a pulled-glass micropipette tip. The coordinates were 0.5, 1.0, and 1.3 mm posterior to the bregma, 1 mm lateral to the bregma, and 0.5 mm deep to the cortical surface.

### Quantification

To quantify axonal sprouting and regeneration in the CST, we obtained serial transverse sections of the spinal cord and quantitatively analyzed the axonal distribution. Fiber sprouting from the white matter of the main CST into the gray matter was quantitatively analyzed via fluorescence microscopy (BX51; Olympus, Tokyo, Japan). We counted the number of BDA-positive fibers in serial transverse sections of the spinal cord (C3–T1; 15 sections per spinal cord segment). The value was normalized to the number of main CST fibers in the most rostral part of C3. The number of BDA-positive fibers in the dorsal CST was determined from images captured by a laser scanning confocal microscope (FV-1200; Olympus) equipped with the ImageJ software (National Institutes of Health, Bethesda, MD, USA).

The lesion depth from the dorsal surface of the spinal cord was measured in sagittal sections containing the lesion epicenter, which was immunostained with anti-GFAP antibody.

### Tissue preparation for histological analysis

Mice were transcardially perfused with PBS followed by 4% paraformaldehyde in 0.1 M phosphate buffer. The spinal cords were dissected, postfixed in the same fixatives, and immersed overnight in PBS containing 30% sucrose, following which they were embedded in Tissue-Tek OCT and frozen at − 80 °C until use. The sections were cut on a cryostat (20 µm-thickness) and mounted on Matsunami adhesive-coated slides (Matsunami, Osaka, Japan). Cryostat sections were incubated with blocking solution containing 5% bovine serum albumin and 0.1–0.3% Triton X-100 in PBS for 1 h at room temperature, followed by overnight incubation with the indicated primary antibodies at 4 °C. Immunoreactivity was visualized using fluorescence-conjugated secondary antibodies. The samples were then coverslipped with mounting medium (Dako) and examined under a fluorescence microscope (Olympus BX53, DP71).

### Immunohistochemistry

Spinal cord sections were prepared 3 days after SCI and brain sections were prepared 14 days after SCI. The sections were immunostained using the following antibodies: anti-GFAP antibody (Dako), anti-myeloperoxidase antibody (Thremo Scientific or Abcam, Cambridge, UK), anti-CD68 antibody (AbD Serotec, Oxford, UK), anti-NeuN antibody, and anti-acetyl-histone H3 antibody (Millipore), alexa 488- or 568-conjugated secondary antibodies (Molecular Probes, Eugene, OR, USA). Alexa 488-conjugated streptavidin was used for the visualization of BDA. The lesion depth from the dorsal surface of the spinal cord was measured following immunostaining with the anti-GFAP antibody.

### Western blot analysis and cytokine array

The motor cortex and the lesion site of the spinal cord tissues were collected on the indicated days after SCI. Tissue lysates were prepared in NP-40 lysis buffer (50 mM Tris-HCl (pH 7.4), 150 mM NaCl, 0.5% NP-40, 10 mM NaF, 1 mM Na3VO4) containing a protease inhibitor cocktail (Roche Diagnostics) at 4 °C, subjected to SDS-PAGE, and electrotransferred to polyvinylidene difluoride membranes (Millipore, Bedford, MA, USA). After blocked with 5% Skim milk in the PBS-T (0.05% Tween-20) for 60 min, membranes were incubated with anti-acetylated histone H3 or anti-histone H3 antibody at 4 °C overnight. Enhanced chemiluminescence plus reagents (GE Healthcare) were used for detection. For cytokine array, mouse cytokine array panel A (R&D Systems, Minneapolis, MN, USA) was used. The spinal cord tissues were prepared 3 days after SCI, and tissue lysates were incubated with a cytokine array membrane according to manufacturer’s protocol. The signals were analyzed using an Amersham imager system (GE Healthcare) or ChemiDoc Touch MP (Bio-Rad, Richmond, CA, USA). The signal intensity was measured using the Image Lab software (Bio-Rad).

### HDAC activity assay

HDAC activity was measured using immunoprecipitation-based HDAC activity assay kit, following the manufacturer’s method (BioVision, Mountain View, CA, USA). The motor cortex and the lesion site of spinal cord tissues were collected after 3 or 14 days after SCI. The tissue extracts were subjected to immunoprecipitation using antibodies for HDAC1, 2, 3, and 8. The immunoprecipitated complex mixed with HDAC substrate, and the activity was measured using plate reader (Molecular Devices, Sunnyvale, CA, USA).

### RNA extraction, reverse transcription, and real-time PCR

Total RNA was extracted from motor cortex samples using TRIzol (Invitrogen) and reverse transcribed using the High-Capacity cDNA Reverse Transcription Kit (Applied Biosystems, Foster City, CA, USA). Real-time PCR was used to determine mRNA expression (QuantStudio 7 Flex Real-time PCR system; ThermoFisher scientific). SYBR Green assay was performed to quantify HDAC expression. Primers for mouse HDACs were designed as previously described^45–47^. A total of 10 μl were used for SYBR green assays, which contained a 1 × final concentration of Fast SYBR green master mix (ThermoFisher Scientific), 400 nM gene-specific primers, and 1 μl template. The PCR cycles were initiated with an uracil *N*-glycosylase digestion stage at 50 °C for 2 min and an initial denaturation period at 95 °C for 10 min, followed by 42 cycles at 95 °C for 15 s, annealing at 60 °C for 1 min, and a gradual increase in temperature from 60 to 95 °C during the dissociation stage. Relative mRNA expression was normalized to the amount of 18 S rRNA in each sample. Cycle threshold values (Ct values) were calculated via the ΔΔCt method to obtain fold differences.

### Cell preparation for FACS analysis

Cells were isolated using modified methods, as previously described^48^. In brief, mice were transcardially perfused with ice-cold PBS, and the spinal cord segments including the lesion epicenter (T3–-T13) were immediately dissected and placed in the ice-cold Hank’s balanced salt solution (Life Technologies) containing collagenase D (2.5 mg/ml; BD Biosciences) and DNase I (0.25 mg/ml; Sigma-Aldrich). After cutting into small pieces, the spinal cord was incubated for 45 min in a 37°C water bath with intermittent shaking. Tissues were homogenized mechanically and passed through a 70-mm cell strainer (BD Biosciences). The collected cell suspension was centrifuged at 700 g for 5 min at room temperature. The pellet was re-suspended in 30% Percoll (GE health care) solution and then slowly loaded on the 70% Percoll solution. Density gradient centrifugation was performed at 1000 g for 25 min at room temperature and the thick myelin-containing layer was removed. Cells were collected from interface of the 30 and 70% Percoll gradient. The cell–Percoll suspension was diluted at least threefold with ice-cold PBS and centrifuged at 1000 g for 10 min at 4°C. Finally, the cells were washed twice with 2 ml of flow cytometry staining buffer (BD Biosciences) for cell surface staining and flow cytometry analysis.

### Cell surface staining and flow cytometry analysis

Anti-mouse CD45-PerCP (BioLegend), anti-mouse CD11b-PE (BioLegend), anti-mouse Ly6C-FITC (BioLegend), and anti-mouse Ly6G-APC antibodies (BioLegend) were used for cell surface staining. The cells were suspended in flow cytometry staining buffer (BD Biosciences) and treated with Fc-receptor blocker (anti-mouse cluster of differentiation 16/32 (CD16/32) antibody (BioLegend) for 20 min at 4 °C in order to eliminate nonspecific binding of antibodies to Fc receptors. Next, the cells continued to be incubated with targeting antibodies for 30 min in the dark. The cells were washed twice with 2 ml of flow cytometry staining buffer and the pellets were finally re-suspended in 500 μl of the staining buffer for detection. Flow cytometry was performed using a BD FACSVerse system (BD Bioscience). The specificity of the antibody signals against specific antigens was determined by performing control experiments using isotype-matched immunoglobulins (BioLegend).

### Statistical analysis

Statistical analyses are described in the figure legends. The data are presented as the mean ± S.E. of at least three independent experiments, and *p* values of < 0.05 were considered to be significant.
